# Finding the molecular scaffold of nuclear receptor inhibitors through high-throughput screening based on proteochemometric modelling

**DOI:** 10.1186/s13321-018-0275-x

**Published:** 2018-04-12

**Authors:** Tianyi Qiu, Dingfeng Wu, Jingxuan Qiu, Zhiwei Cao

**Affiliations:** 1 School of Life Sciences and Technology, Shanghai 10th People’s Hospital, Tongji University, No. 1239 SiPing Road, Shanghai, China; 2 The Institute of Biomedical Sciences, Fudan University, No. 138 Medical College Road, Shanghai, China; 3 School of Medical Instrument and Food Engineering, University of Shanghai for Science and Technology, No. 516 JunGong Road, Shanghai, China

**Keywords:** Proteochemometric modelling, Nuclear receptor, Molecular scaffold, Cheminformatics

## Abstract

Nuclear receptors (NR) are a class of proteins that are responsible for sensing steroid and thyroid hormones and certain other molecules. In that case, NR have the ability to regulate the expression of specific genes and associated with various diseases, which make it essential drug targets. Approaches which can predict the inhibition ability of compounds for different NR target should be particularly helpful for drug development. In this study, proteochemometric modelling was introduced to analysis the bioactivity between chemical compounds and NR targets. Results illustrated the ability of our PCM model for high-throughput NR-inhibitor screening after evaluated on both internal (AUC > 0.870) and external (AUC > 0.746) validation set. Moreover, in-silico predicted bioactive compounds were clustered according to structure similarity and a series of representative molecular scaffolds can be derived for five major NR targets. Through scaffolds analysis, those essential bioactive scaffolds of different NR target can be detected and compared. Generally, the methods and molecular scaffolds proposed in this article can not only help the screening of potential therapeutic NR-inhibitors but also able to guide the future NR-related drug discovery.

**Electronic supplementary material:**

The online version of this article (10.1186/s13321-018-0275-x) contains supplementary material, which is available to authorized users.

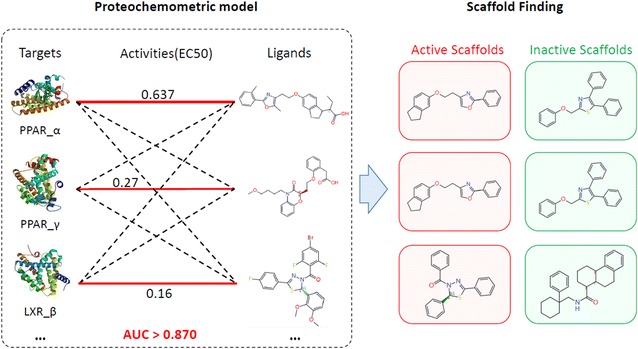

## Background

As a ligand dependent transcription factors, nuclear receptors (NR) can be activated by important molecules such as steroidal hormones, endogenous hormones, glucocorticoids and thyroid hormones [1, 2]. After activation, NR can regulate the expression of specific genes and then participate in several essential physiological processes such as development, homeostasis and metabolism of the organism [1, 2]. Since NR can affect the expression of enormous genes which associated with various diseases such as diabetes and hepatic adipose infiltration, it can be considered as an appropriate therapeutic target for new drug discovery. Till now, 48 nuclear receptors have been discovered in humans [3], 23 of them are certified as drug target by U.S. Food and Drug Administration (FDA). Meanwhile, over 13% FDA approved drugs were aimed at those nuclear receptors [4]. In that case, discover novel drugs as nuclear receptor inhibitors have acquired a particular significance for NR-related metabolic diseases treatment. In drug design, scaffold is the fixed part of a molecule which is the essential part for biological activity of molecule. Therefore, scaffold based strategies were widely used for drug discovery [5–7]. It can be noticed that finding a new scaffold often lead to the discovery of a new inhibitor classes which may have the potential to become future drugs [8–10]. In that case, finding novel bioactive scaffolds is an essential process in the area of drug design.

In order to discover the molecular scaffold of a class of molecules such as NR-inhibitors, massive structure of molecules with bioactivity need to be screened and clustered to finding the consensus structure domain. Traditionally, this screening evolving titration experiments is a time-consuming, expensive and labor-intensive process, which could be assisted by computer-aided drug design (CADD) [11]. In recent decades, different methods including virtual screening [12, 13], molecular docking [14, 15], de-novo drug design [16–18], pharmacophore modeling [19–21] and molecular dynamics [22, 23] were introduced to find bioactive molecules for further drug design. In the early 1960 s, quantitative structure activity relationship (QSAR) approach was established to discover the relationship between ligand and target [24]. In general, conventional QSAR based approaches consider structure information and bio-active value to efficiently predict the relationship between ligand and target. However, its prediction ability is limited to single target and enable to map multiple ligand-target relationship [25]. Also, the prediction ability of conventional QSARs were limited since only ligand information were used for model construction [25–27]. To avoid the shortages of QSAR, an approach relying on the description of both ligand and target to quantitatively analyze their relations was invented and termed as Proteochemometric (PCM) modeling in 2001 [28]. The main advantage of PCM modeling is to integrate information on both ligand and target to make the model applicable for multiple target screening, including GPCRs [29–31], proteases [32–34], kinases [35, 36], reverse transcriptase [37, 38]. However, according to author’s knowledge, PCM for NR-inhibitor prediction was hardly reported.

In this article, two major steps including PCM modelling and scaffold finding were processed to guide the design of NR-inhibitors. Initially, based on a total number of 11 nuclear receptors and 9633 molecular compounds with EC_50_ values were derived from ONRLDB [39], a series of PCM modelling were generated to predict the inhibition ability for NR-inhibitors. After rigorous validation through both internal and external validation dataset, our PCM model was proved to have the potential ability for high-throughput NR-inhibitor screening. It should be noted that NR-targets validated in external dataset were not involved in our training set. That means for those NR proteins without enough bio-active data to establish a traditional QSAR models, our model may also have the ability to provide NR-inhibitor screening. Further, after molecular clustering based on our PCM model, novel bioactive scaffolds for NR-inhibitors can be discovered. The potential bioactive scaffolds for different NR targets were proposed for future drug discovery of NR-inhibitors.

## Results and discussion

### Construction of proteochemometric modeling

To build a proteochemometric modeling, three parts are necessarily needed: (1) bio-active data between multiple compounds and multiple targets; (2) descriptors which includes both ligand and target information; and (3) suitable learning methods to link descriptors and bio-active data. Here, for model construction, bio-active data with the most strict cutoff of EC_50_ = 1 μm was chosen as classification indicator. Then, to test the performance of different target descriptors, both sequence similarity descriptor and structure similarity descriptor were tested and four types of descriptors marked as T1–T4 were generated in this study (see “Methods” Part). Further, five different machine learning approaches including Random Forest (RF), Ridge Classifier (RC), Logistic Regression (LR), Decision Tree (DT) and Support Vector Classification (SVC) were used to establish different PCM models. Through 10-fold cross-validation, the performance of all five machine learning algorithms shows that Random Forest classifier can obtains the best prediction performance with the highest accuracy over 0.73 among all five and followed by Decision Tree (Table 1 and Additional file 1: Table S1). The AUC (area under curve) value also indicated that Random Forest classifier can achieves better prediction abilities than others for select NR-inhibitors. Therefore, Random Forest classifier was chose to establish our PCM modeling.

**Table 1:** 10-fold cross-validation results of different machine learning methods

Method	Accuracy	Precision	Recall	F1_score	AUC
RF	0.740	0.761	0.768	0.762	0.829
RC	0.624	0.643	0.713	0.674	–^a^
LR	0.453	0.490	0.000	0.000	0.452
DT	0.701	0.726	0.727	0.726	0.700
SVC	0.583	0.569	0.984	0.720	–^a^

Results in Table 1 were calculated based on descriptor T1

^a^This parameters can’t be calculated in here (continuous predict values are needed to calculate AUC value)

**Fig. 1:**
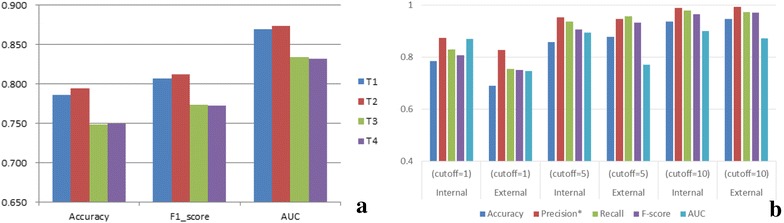
Performance of PCM modeling. **a** Cross-validation performance of PCM model constructed by RF classifier based on four different protein descriptors. **b** AUC value of PCM modeling constructed by RF classifier under different cutoffs of bio-active data, this results were obtained by descriptor T1. *Precision score means the area under the precision-recall curve

After that, the performances of 4 different descriptors were also tested by RF classifier (Fig. 1a). Results showed that the performance of sequence descriptors and structure descriptors are quite similar, which may cause by the fact that the structure feature of NR family is highly conserved. However, the performance of T1 and T2 is significantly better that those of T3 and T4, that means protein descriptors based on background data of whole protein family can better describe the properties of target proteins in model. And further, these protein descriptors can be extended to other proteins in the NR families. Considering that structure descriptors requires crystal structures which may not be available for several targets, in this study, sequence descriptors based on 30 background protein (T1) were used for model construction.

Further, the contribution of chemical descriptor was also analyzed. After statistic analysis, it can be found that lipo-hydro partition coefficient (MolLogP in RDKit) contains the major contribution among all ligand descriptors, which means it might be the key element for molecular with potential inhibition abilities (Additional file 2: Fig. S1). It can also found that, for both active compound and inactive compound, the distribution of MolLogP follows Normal distribution with significant difference, which were calculate through *T* test (P value < 0.0001). Result showed that, lipo-hydro partition coefficient is important for the activity of NR inhibitor, active compounds normally contain MolLogP around 5.775, while the MolLogP of inactive compounds were around 5.380. Importance and P value of top 10 chemical structure descriptors can be found in Additional file 3: Table S2.

### Evaluation of proteochemometric modeling

In this study, PCM modeling was systemically evaluated through both internal and external validations. By setting different cutoffs of bio-active data, results of different PCM models can be found in Fig. 1b, detailed information of model performance on all four protein descriptors can be found in Additional file 4: Table S3. Generally, all PCM models can gives outstanding performance in internal validation by achieving an AUC value above 0.870 on different cutoffs. For external validation, all PCM model can also achieves a satisfied performance with AUC value over 0.746. Above results indicate the excellent ability of our PCM model for NR-related inhibitors prediction. Also, with the increasing of cutoffs, the performance of PCM models increased synchronously. This probably caused by the fact that the unbalance between positive and negative data according to different cutoff. For example, when set EC_50_ ≤ 1 as positive data and EC_50_ > 1 as negative data, the ratio (positive/negative) of training set, testing set and external validation set were all close to 1 (Additional file 5: Table S4). After the cutoff rising to 10, those ratios were quickly increased to 12.14, 12.95 and 22.76 respectively (Additional file 5: Table S4). Several reports also pointed out that the 1 μM cutoff may be more reasonable because it contains less noise [40]. In that case, the cutoff of EC_50_ value was set as 1 for further analysis.

### Finding the molecular scaffolds for NR inhibitors

**Fig. 2:**
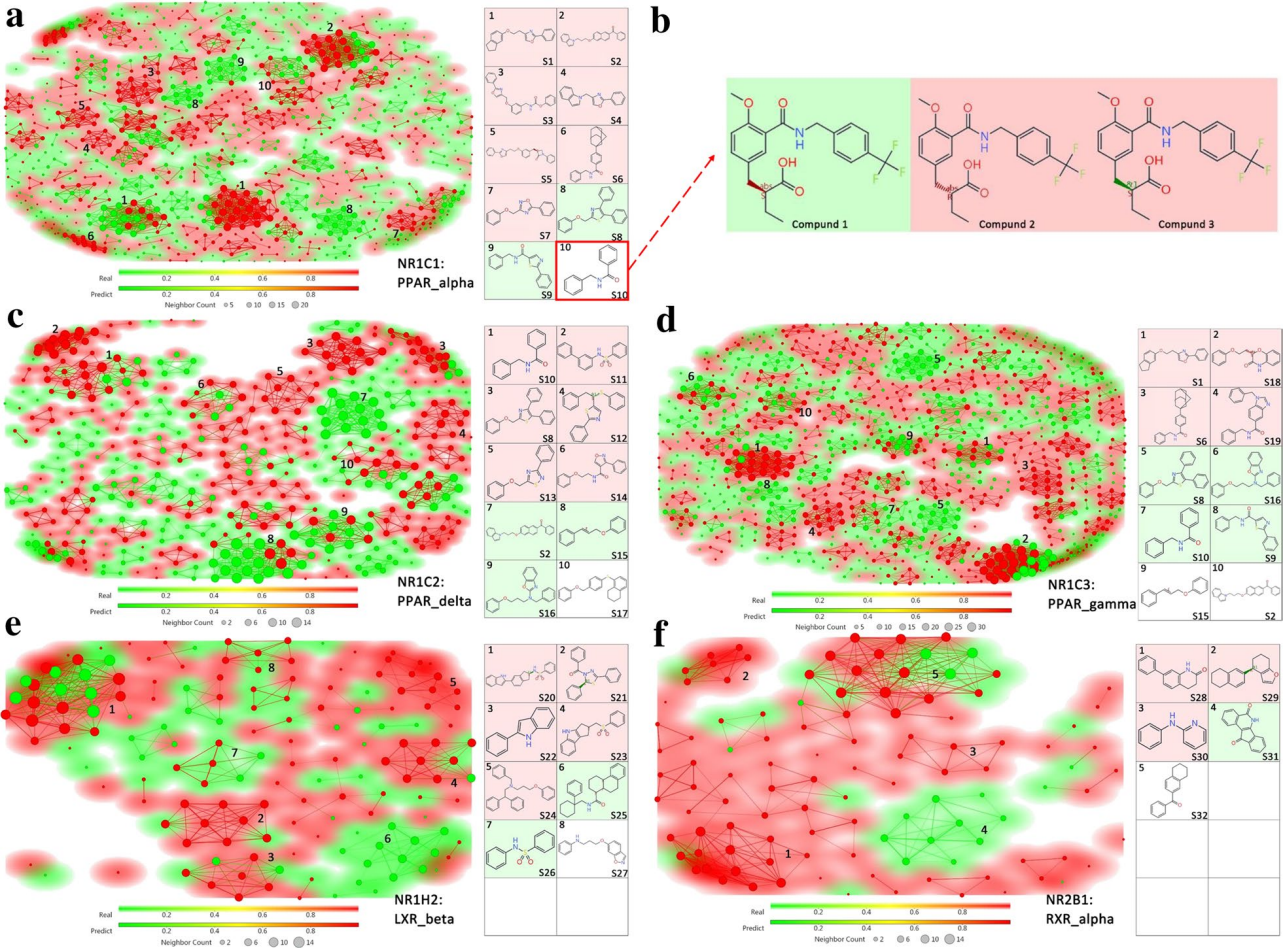
Scaffold clustering of NR-inhibitors, colors in back ground and in spot represents the experimental confirmed active compounds and model predicted active compounds respectively. Red means active compounds while green means inactive compounds, white color means scaffold contains both active and inactive compounds. **a** Scaffold clustering of NR1C1-inhibitors. **b** Examples of compounds contains scaffold S10. **c** Scaffold clustering of NR1C2-inhibitors. **d** Scaffold clustering of NR1C3-inhibitors. **e** Scaffold clustering of NR1H2-inhibitors. **f** Scaffold clustering of NR2B1-inhibitors

To further validate our PCM model, the active and inactive inhibitors were predicted through our PCM model. Then, the Rubberbanding Forcefield approach in DataWarrior [41] (release version 4.5.2) was used to mapping all compounds into a 2-dimentional area, while similar molecules were located close to each other (see “Molecular scaffold searching”). In that case, molecules with structure similarity over 0.95 will be clustered together. The molecules clustering and corresponding scaffolds for top clusters were illustrated in Fig. 2. Chemical name and smiles files of corresponding scaffolds were listed in Additional file 6: Table S5. Background color mapping of different NR proteins were derived from experimental values. Red color in background means active clusters while green ones means inactive clusters. Each spot represents one compound in our testing set, which were classified by our model. Red spot represents active compounds while green spot means inactive ones. The location of each compound determined by structure similarity, compounds with similar structures tend to clustered together. Compounds with similarity over a certain threshold will be defined as neighbors and connected with lines. The size of each compound spot is related to the number of its neighbor spots.

Generally, the prediction of our PCM model matched perfectly well with the experimental values. For three peroxisome proliferator-activated receptor (PPAR) protein targets, the top 10 clusters of each target including NR1C1 (Fig. 2a), NR1C2 (Fig. 2c) and NR1C3 (Fig. 2d) were detected and marked in each sub-graphs. For PPAR protein targets, both unique and overlapped scaffolds can be detected. For example, target NR1C1 contains 7 bioactive scaffolds (marked as S1 to S7), 2 inactive scaffolds (marked as S8 and S9) and 1 mixed scaffold (marked as S10) contains both active and inactive compounds. Among above, scaffold S1 and S6 were active in both NR1C1 and NR1C3 (Fig. 2d), while S8 and S9 were both inactive scaffold. On the other hand, different pattern can be found in target NR1C2 (Fig. 2c). In NR1C2, 7 new scaffold clusters marked as S11 to S18 were detected. Besides that, as a major inactive scaffold for NR1C1 and NR1C3, S8 was determined as active scaffold in NR1C2. Also, as an active scaffold in NR1C1 and mixed scaffold in NR1C3, scaffold S2 was defined as inactive scaffold for NR1C2. The results of two targets beside PPAR targets were quite different, totally new scaffolds were discovered and illustrated in Fig. 2e, f. All above illustrated that, even from the same protein family, the inhibitor scaffolds of different NR protein targets were still distinguishable.

Also, it should be noticed that, the bioactivity of different compounds rely on multiple factors such as side-chain composition, functional group, substituent and chirality. For instance, scaffold S10 N-benzylbenzamide contains different compounds including compound 1–3 (Fig. 2b). The molecular structure of three compounds is extremely similar except for the chirality. The stereogenic center of compound 1 (Benzenepropanoic acid, α-ethyl-4-methoxy-3-[[[[4-(trifluoromethyl)phenyl]methyl]amino]carbonyl]-, (αS)-) and compound 2 (Benzenepropanoic acid, α-ethyl-4-methoxy-3-[[[[4-(trifluoromethyl)phenyl]methyl]amino]carbonyl]-, (αR)-) are absolutely configured as S and R, respectively. Compound 3 was defined as mixture of stereoisomers which may combine with both S and R chirality.

## Discussion

Computer-aided drug design (CADD) can assist and shorten the process of new drug discovery. To achieve that, one essential issue is to per-estimate the activity of different compound against different target proteins. By introducing PCM model into CADD, relationship between multiple compounds and targets can be determined. Based on high-throughput screening of compounds, bioactive molecules can be clustered and essential molecular scaffolds can be detected to guide the future development of therapeutic drugs.

In order to process high-throughput screening of bioactive inhibitors for targets from NR families, 7267 bio-active data of 11 nuclear receptors were collected to establish an in silico model. Through both internal and external validation, our PCM models were proved to be sensitive for NR-inhibitor prediction which might be benefit from our descriptors. For target descriptors, generalized sequence similarity descriptors contain information from 30 background targets from NR families. Models based on those descriptors can achieve a better prediction performance on both internal and external validation set, which means those descriptors can be extended to multiple targets from NR families. For chemical descriptors, since lipo-hydro partition coefficient contains the major contribution for classification and parameter MolLogP is distinguishable for active and inactive compounds, this may provide a clue for future therapeutic NR-inhibitors discoveries.

Another essential issue for PCM model construction is to choose the suitable machine learning method. In this study, five different machine learning methods including both regression and classification approaches were tested to establish PCM modeling. Results showed that the performance of RF and DT classifier are significantly higher than other methods, which means above algorithms might be more applicable in the case of NR-inhibitors prediction.

After high-throughput screening of NR-inhibitors, bioactive molecules could be clustered according to structure similarity and molecular scaffold enriched in each clustered can be detected and might assist the process of drug design. In this article, the appropriate models selected after evaluations were used to molecular clustering for five major NR targets. Results showed that our PCM model can successfully predict those potential NR-inhibitors which agree well with the experimental EC_50_ values. For each NR target, our algorithms can able to predict those potential therapeutic inhibitors and discover the molecular scaffolds for future drug development. Currently, this method was established on NR proteins and it can be extended to other protein targets after the accumulating of experimental data.

## Methods

### Data set

**Fig. 3:**
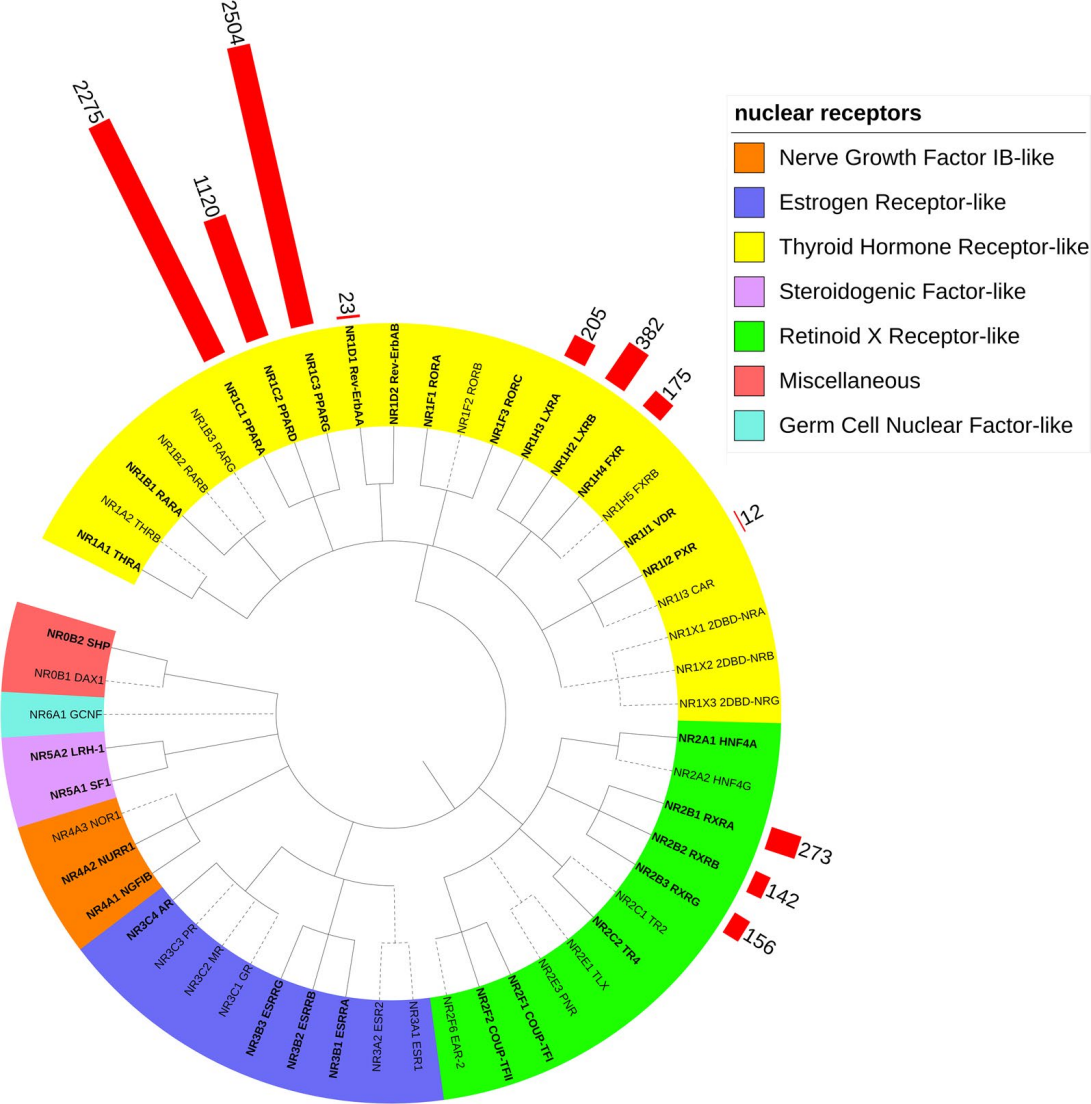
Clustering tree of nuclear receptors. 7 different subtypes of NR were marked in different colors and 11 NR proteins used in this study were marked in red as well as its data distribution

Training and validation dataset of nuclear receptor and its inhibitors were collected from ONRLDB [39], which including information of 11 protein targets and 9633 molecular compounds (see Additional file 7: information of 9633 compounds.sdf). After filtration, a total number of 7267 inhibitors for 11 nuclear receptors with half maximal effective concentration (EC_50_) values were remained as our dataset. After primary statistic analyze, it can be found that the distribution of bio-active data for each protein targets were unbalanced (Fig. 3 and Additional file 8: Table S6). Major target contains more than thousands of bioactive data while several only covering tens of data. Five major protein target including NR1C1, NR1C2, NR1C3, NR1H2, NR2B1 contains over 90% of the bio-active data, which provide an abundant data for model construction. The remaining 6 targets (NR1H3, NR1H4, NR2B3, NR2B2, NR1D1, and NR1I2) with bio-active data were selected as independent validation set. After above steps, internal dataset which including 6554 bio-active data with corresponding protein targets (5 major nuclear receptor) and compounds were selected for Proteochemometric modeling. For each target, 60% of the bio-active data were chosen as training set and the rest remained as testing set. In general, 3931 bio-active data were selected as training set to generate our PCM model and the rest 2623 were used for model evaluation. Besides that, 713 bio-active data for other 6 NR proteins were collected as external validation dataset. Further, 30 crystal structures of NR from different sub-type with highest resolution were selected as background NR target (Additional file 9: Table S7).

### Protein target descriptor

Here, both sequence similarity descriptors and structure similarity descriptors were used to characterize those five nuclear receptors. Firstly, a 30 protein targets from NR families can be derived from Protein Data Bank (PDB) [42] as background. For 11 protein targets in our dataset, the sequence and structure similarity compared with those 30 background protein target structures can be calculated by pairwise alignment respectively. Sequence alignment was calculated by smith-waterman alignment [43], while structure alignment was calculated by using jFATCAT [44]. Therefore, two types of generalized target descriptor including sequence similarity descriptor (T1) and structure similarity descriptor (T2) can be obtained for each protein targets. For comparison, specific descriptors based on 5 protein target from our training set instead of 30 background protein target were also established, recorded as T3 (specific sequence similarity descriptor based on 5 protein target) and T4 (specific structure similarity descriptor based on 5 protein target). Two generalized target descriptors can be found in Additional file 10: Table S8-1, 2 and two specific target descriptors were also listed in Additional file 11: Table S9-1, 2.

### Inhibitor descriptor

Chemical structure descriptors were calculated by using RDKit (release version 2016). RDkit provides different chemical structure descriptors, which contains both chemical and physical properties such as Molecular Weight, Hydrogen Bond Donor Count, Hydrogen Bond Acceptor Count, Rotatable Bond Count and LogP etc. In addition, RDKit contains massive types of chemical descriptors derived from other tools and literatures, such as MOE-type descriptors for partial charges, MR contributions, LogP contributions, EState indices and surface area contributions integrated from molecular operating environment (MOE). In general, 187 descriptors were used to characterize the structure features of inhibitor (Additional file 12: Table S10).

### Proteochemometric modeling

In this study, 4 Proteochemometric models were created from training set based on different combinations of descriptors (T1-L, T2-L, T3-L, T4-L). All models were implemented in scikit-learn (Version 0.18.1) by using Random Forest (RF) with default parameters. For classification, different thresholds of EC_50_ were selected to distinguish positive and negative data. Here, three different thresholds (EC_50_ < 1 μm, EC_50_ < 5 μm and EC_50_ < 10 μm) were used for classification respectively.

### Model evaluation

For each combination of descriptors, 10-fold cross-validation was carried out for the model. The performance of four models was assessed by classification accuracy. Further, both internal and external validation data were tested from different aspects to evaluate the overall performance of our models, including the area under the ROC curve (AUC) value, accuracy, precision, recall and F-score, statistical parameters were defined in the following equations:

1 $${\text{Accuracy}} = \frac{TP + TN}{TP + FP + TN + FN}$$

2 $${\text{Precision}} = \frac{\text{TP}}{{{\text{TP}} + {\text{FP}}}}$$

3 $${\text{Recall}} = \frac{TP}{TP + FN}$$

4 $${\text{F-score}} = 2 \cdot \frac{precision \cdot recall}{precision + recall}$$

Positive samples are those with EC_50_ value below threshold. TP represents True positive, TN represents True negative, FP represents false positive and FN represent false negative.

### Molecular scaffold searching

For each protein target, the similarity of corresponding molecules were analyzed based on *Rubberbanding Forcefield* approach in DataWarrior [41] (release version 4.5.2). Initially, all molecules were translated into a series of descriptors to encode various aspects of chemical structures including both 2-D and 3-D structure information. After that, calculate the entire similarity matrix between all molecules and locate most similar neighbors to be considered for every molecules. Then, stepwise relocate all molecules to ensure similar molecules were located close to each other. Finally, molecules with structure similarity over 0.95 will be clustered together [41]. For each cluster, the major Bemis-Murcko scaffold [45] (covering over 80% of the molecules in this cluster) was defined as the representative scaffold. Note that for several clusters, no major scaffold can be detected, in that case, the maximum common substructures for each two scaffolds can be calculated through RDKit and the major substructure was defined as the representative scaffold. After that, the Bemis-Murcko scaffold for each cluster can be derived and analyzed.

## Additional files

**Additional file 1: Table S1.** 10-fold cross-validation results of different machine learning methods on four descriptors.

**Additional file 2: Fig. S1.** Distributions of MolLogP in both active compound and inactive compound.

**Additional file 3: Table S2.** Importance and P value of top 10 chemical structure descriptors.

**Additional file 4: Table S3.** Model performance of random forest classifier on four protein descriptors.

**Additional file 5: Table S4.** Data distribution of training set, testing set and external validation set.

**Additional file 6: Table S5.** Chemical name and smiles file of selected scaffold.

**Additional file 7.** Supplementary Data 1: information of 9633 compounds.

**Additional file 8: Table S6.** Data distribution of different NR targets.

**Additional file 9: Table S7.** Information of crystal structure used for descriptor generation.

**Additional file 10: Table S8-1.** Sequence similarity descriptors based on 30 NR proteins (T1). **Table S8-2.** Structure similarity descriptors based on 30 NR proteins (T2).

**Additional file 11: Table S9-1.** Sequence similarity descriptors based on 5 NR proteins (T3). **Table S9-2.** Structure similarity descriptors based on 5 NR proteins (T4).

**Additional file 12: Table S10.** Information of inhibitor descriptors.
