# Supplementary material for: Finding the molecular scaffold of nuclear receptor inhibitors through high-throughput screening based on proteochemometric modelling
Source: J Cheminform. 2018 Apr 12;10:21. doi: 10.1186/s13321-018-0275-x (PMC5897275; doi:10.1186/s13321-018-0275-x)
Supplement: Supplementary file 1 — Additional file 1: Table S1. 10-fold cross-validation results of different machine learning methods on four descriptors. [file 13321_2018_275_MOESM1_ESM.docx]

Additional file 1: Table S1. 10-fold cross-validation results of different machine learning methods on four descriptors.

| cutoff | 1* | | | | 5 | | | | 10 | | | |
| --- | --- | --- | --- | --- | --- | --- | --- | --- | --- | --- | --- | --- |
| Descriptor | T1 | T2 | T3 | T4 | T1 | T2 | T3 | T4 | T1 | T2 | T3 | T4 |
| RF | **0.740** | **0.738** | **0.727** | **0.738** | **0.841** | **0.857** | **0.830** | **0.825** | **0.938** | **0.929** | **0.934** | **0.937** |
| RC | 0.624 | 0.616 | 0.615 | 0.635 | 0.747 | 0.761 | 0.762 | 0.748 | 0.930 | 0.933 | 0.928 | 0.928 |
| LR | 0.453 | 0.447 | 0.457 | 0.449 | 0.741 | 0.737 | 0.741 | 0.732 | 0.926 | 0.927 | 0.926 | 0.922 |
| DT | 0.701 | 0.712 | 0.692 | 0.716 | 0.787 | 0.796 | 0.793 | 0.784 | 0.915 | 0.911 | 0.910 | 0.914 |
| SVC | 0.583 | 0.642 | 0.583 | 0.586 | 0.756 | 0.759 | 0.756 | 0.752 | 0.931 | 0.930 | 0.931 | 0.926 |

*Results listed in here represented the prediction accuracy of each machine learning methods on four descriptors.
