# Supplementary figures and images for: Finding the molecular scaffold of nuclear receptor inhibitors through high-throughput screening based on proteochemometric modelling
Source: J Cheminform. 2018 Apr 12;10:21. doi: 10.1186/s13321-018-0275-x (PMC5897275; doi:10.1186/s13321-018-0275-x)

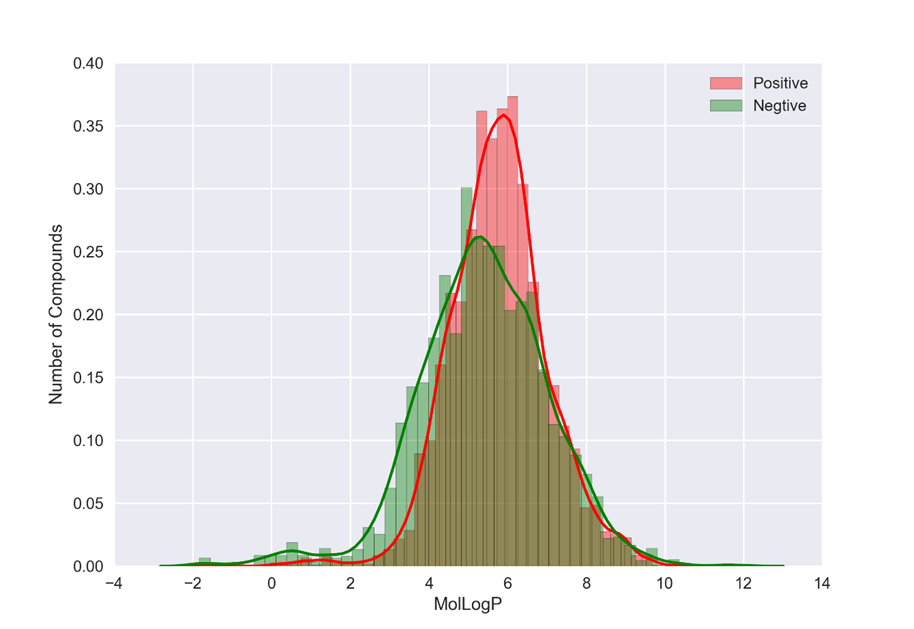


Additional file 2: Fig. S1. Distributions of MolLogP in both active compound and inactive compound

Supplement: Supplementary file 2 — Additional file 2: Fig. S1. Distributions of MolLogP in both active compound and inactive compound. [file 13321_2018_275_MOESM2_ESM.docx]
