# Supplementary material for: Finding the molecular scaffold of nuclear receptor inhibitors through high-throughput screening based on proteochemometric modelling
Source: J Cheminform. 2018 Apr 12;10:21. doi: 10.1186/s13321-018-0275-x (PMC5897275; doi:10.1186/s13321-018-0275-x)
Supplement: Supplementary file 3 — Additional file 3: Table S2. Importance and P value of top 10 chemical structure descriptors. [file 13321_2018_275_MOESM3_ESM.docx]

Additional file 3: Table S2. Importance and P-value of top 10 chemical structure descriptors.

| **Descriptor** | **Importance**^#^ | **p-value** |
| --- | --- | --- |
| **MolLogP** | 0.026 | 2.946E-29 |
| **MaxAbsEStateIndex** | 0.020 | 1.970E-08 |
| **MinAbsEStateIndex** | 0.019 | 2.783E-10 |
| **VSA_EState8** | 0.019 | 1.532E-07 |
| **SMR_VSA5** | 0.019 | 9.350E-35 |
| **MinEStateIndex** | 0.018 | 2.039E-03 |
| **BalabanJ** | 0.017 | 5.517E-03 |
| **MaxEStateIndex** | 0.017 | 5.517E-03 |
| **BertzCT** | 0.016 | 2.243E-07 |
| **FractionCSP3** | 0.016 | 1.901E-15 |
| **SMR_VSA9** | 0.016 | 4.921E-05 |
| **Kappa1** | 0.015 | 3.343E-17 |
| **Chi2v** | 0.015 | 1.599E-23 |
| **PEOE_VSA8** | 0.015 | 2.437E-10 |
| **VSA_EState9** | 0.015 | 4.601E-01 |

^#^Importance represents the contribution of the matching feature to the prediction function.
