# Supplementary material for: Finding the molecular scaffold of nuclear receptor inhibitors through high-throughput screening based on proteochemometric modelling
Source: J Cheminform. 2018 Apr 12;10:21. doi: 10.1186/s13321-018-0275-x (PMC5897275; doi:10.1186/s13321-018-0275-x)
Supplement: Supplementary file 4 — Additional file 4: Table S3. Model performance of random forest classifier on four protein descriptors. [file 13321_2018_275_MOESM4_ESM.docx]

Additional file 4: Table S3. Model performance of random forest classifier on four protein descriptors.

|  | Cutoff=1 | | Cutoff=5 | | Cutoff=10 | |
| --- | --- | --- | --- | --- | --- | --- |
| T1 | Test | Extend | Test | Extend | Test | Extend |
| Accuracy | 0.79 | 0.69 | 0.86 | 0.88 | 0.94 | 0.95 |
| Precision | 0.87 | 0.83 | 0.95 | 0.95 | 0.99 | 0.99 |
| Recall | 0.83 | 0.75 | 0.94 | 0.96 | 0.98 | 0.97 |
| F1_score | 0.81 | 0.75 | 0.91 | 0.93 | 0.97 | 0.97 |
| AUC | 0.87 | 0.75 | 0.89 | 0.77 | 0.90 | 0.87 |
|  |  |  |  |  |  |  |
| T2 | Test | Extend | Test | Extend | Test | Extend |
| Accuracy | 0.79 | 0.69 | 0.85 | 0.87 | 0.94 | 0.95 |
| Precision | 0.88 | 0.82 | 0.95 | 0.95 | 0.99 | 0.99 |
| Recall | 0.82 | 0.74 | 0.94 | 0.94 | 0.98 | 0.97 |
| F1_score | 0.81 | 0.75 | 0.90 | 0.92 | 0.97 | 0.97 |
| AUC | 0.87 | 0.75 | 0.88 | 0.78 | 0.92 | 0.87 |
|  |  |  |  |  |  |  |
| T3 | Test | Extend | Test | Extend | Test | Extend |
| Accuracy | 0.75 | 0.67 | 0.85 | 0.87 | 0.94 | 0.95 |
| Precision | 0.84 | 0.80 | 0.95 | 0.95 | 0.99 | 0.98 |
| Recall | 0.79 | 0.77 | 0.93 | 0.94 | 0.99 | 0.98 |
| F1_score | 0.77 | 0.74 | 0.90 | 0.93 | 0.97 | 0.97 |
| AUC | 0.83 | 0.74 | 0.88 | 0.77 | 0.93 | 0.70 |
|  |  |  |  |  |  |  |
| T4 | Test | Extend | Test | Extend | Test | Extend |
| Accuracy | 0.75 | 0.69 | 0.85 | 0.84 | 0.94 | 0.94 |
| Precision | 0.84 | 0.83 | 0.94 | 0.95 | 0.99 | 0.99 |
| Recall | 0.78 | 0.76 | 0.91 | 0.91 | 0.98 | 0.98 |
| F1_score | 0.77 | 0.75 | 0.90 | 0.91 | 0.97 | 0.97 |
| AUC | 0.83 | 0.75 | 0.88 | 0.77 | 0.91 | 0.84 |
