# Supplementary material for: Finding the molecular scaffold of nuclear receptor inhibitors through high-throughput screening based on proteochemometric modelling
Source: J Cheminform. 2018 Apr 12;10:21. doi: 10.1186/s13321-018-0275-x (PMC5897275; doi:10.1186/s13321-018-0275-x)
Supplement: Supplementary file 5 — Additional file 5: Table S4. Data distribution of training set, testing set and external validation set. [file 13321_2018_275_MOESM5_ESM.docx]

Additional file 5: Table S4. Data distribution of training set, testing set and external validation set.

|  | Training set | | | Testing set | | | External validation set | | |
| --- | --- | --- | --- | --- | --- | --- | --- | --- | --- |
| Cutoff | active | inactive | ratio | active | inactive | ratio | active | inactive | ratio |
| 1 | 2143 | 1788 | 1.198 | 1423 | 1200 | 1.185 | 440 | 273 | 1.611 |
| 5 | 2914 | 1017 | 2.865 | 1940 | 683 | 2.840 | 616 | 97 | 6.350 |
| 10 | 3632 | 299 | 12.147 | 2435 | 188 | 12.952 | 683 | 30 | 22.766 |
