# Supplementary material for: Finding the molecular scaffold of nuclear receptor inhibitors through high-throughput screening based on proteochemometric modelling
Source: J Cheminform. 2018 Apr 12;10:21. doi: 10.1186/s13321-018-0275-x (PMC5897275; doi:10.1186/s13321-018-0275-x)
Supplement: Supplementary file 6 — Additional file 6: Table S5. Chemical name and smiles file of selected scaffold. [file 13321_2018_275_MOESM6_ESM.docx]

Additional file 6: Table S5. Chemical name and smiles file of selected scaffold.

| ID | Chemical Name | smiles |
| --- | --- | --- |
| S1 | 4-(2,3-dihydro-1H-inden-5-yloxymethyl)-2-phenyl-1,3-oxazole | C1CC2=C(C1)C=C(C=C2)OCC3=COC(=N3)C4=CC=CC=C4 |
| S2 | (6-(3-(1H-indol-1-yl)propoxy)naphthalen-2-yl)(phenyl)methanone | O=C(C4(=CC=3(C(=CC(OCCCN2(C1(=C(C=CC=C1)C=C2)))=CC=3)C=C4)))C5(=CC=CC=C5) |
| S3 | phenyl (3-((2-phenyloxazol-4-yl)methoxy)benzyl)carbamate | O=C(OC1(=CC=CC=C1))NCC4(=CC(OCC=3(N=C(C2(=CC=CC=C2))OC=3))=CC=C4) |
| S4 | 4-((1H-indol-1-yl)methyl)-2-phenyloxazole | O1(C(=NC(=C1)CN3(C2(=C(C=CC=C2)C=C3)))C4(=CC=CC=C4)) |
| S5 | 1-phenyl-3-(3-(2-(2-phenyloxazol-4-yl)ethoxy)benzyl)azetidin-2-one | O=C2(N(C1(=CC=CC=C1))CC2CC5(=CC(OCCC=4(N=C(C3(=CC=CC=C3))OC=4))=CC=C5)) |
| S6 | 4-(adamantan-1-yl)-N-benzylbenzamide | O=C(NCC1(=CC=CC=C1))C5(=CC=C(C23(CC4(CC(C2)CC(C3)C4)))C=C5) |
| S7 | 3-(phenoxymethyl)-5-phenyl-1,2,4-oxadiazole | C1=CC=C(C=C1)C2=NC(=NO2)COC3=CC=CC=C3 |
| S8 | 2-(phenoxymethyl)-4,5-diphenylthiazole | S3(C(C1(=CC=CC=C1))=C(C2(=CC=CC=C2))N=C3COC4(=CC=CC=C4)) |
| S9 | N-benzyl-2-phenyl-1,3-thiazole-5-carboxamide | C1=CC=C(C=C1)CNC(=O)C2=CN=C(S2)C3=CC=CC=C3 |
| S10 | N-benzylbenzamide | C1=CC=C(C=C1)CNC(=O)C2=CC=CC=C2 |
| S11 | N-(2-phenylphenyl)benzenesulfonamide | S(=O)(=O)(NC2(=CC(C1(=CC=CC=C1))=CC=C2))C3(=CC=CC=C3) |
| S12 | 2-phenyl-5-(2-phenyl-1-(phenylthio)ethyl)thiazole | S1(C(=NC=C1C(SC2(=CC=CC=C2))CC3(=CC=CC=C3))C4(=CC=CC=C4)) |
| S13 | 5-(phenoxymethyl)-3-phenyl-1,2,4-thiadiazole | S2(N=C(C1(=CC=CC=C1))N=C2COC3(=CC=CC=C3)) |
| S14 | N-(2-phenoxyethyl)-3-phenylisoxazole-4-carboxamide | O=C(NCCOC1(=CC=CC=C1))C=2(C(=NOC=2)C3(=CC=CC=C3)) |
| S15 | [(E)-3-phenoxyprop-1-enyl]benzene | C1=CC=C(C=C1)C=CCOC2=CC=CC=C2 |
| S16 | N-benzyl-N-(3-phenoxypropyl)benzo[d]oxazol-2-amine | O1(C(=NC2(=C1C=CC=C2))N(CC3(=CC=CC=C3))CCCOC4(=CC=CC=C4)) |
| S17 | (4-(phenoxymethyl)phenyl)(5,6,7,8-tetrahydronaphthalen-1-yl)sulfane | S(C1(=C2(C(=CC=C1)CCCC2)))C4(=CC=C(COC3(=CC=CC=C3))C=C4) |
| S18 | 2-(2-phenoxyethyl)-2H-benzo[b][1,4]oxazin-3(4H)-one | O=C2(NC1(=C(C=CC=C1)OC2CCOC3(=CC=CC=C3))) |
| S19 | N,1-dibenzyl-1H-benzo[d]imidazole-5-carboxamide | O=C(NCC1(=CC=CC=C1))C3(=CC=2(N=CN(C=2C=C3)CC4(=CC=CC=C4))) |
| S20 | N-[5-(1H-indol-2-yl)-2,3-dihydro-1H-inden-2-yl]benzenesulfonamide | C1C(CC2=C1C=CC(=C2)C3=CC4=CC=CC=C4N3)NS(=O)(=O)C5=CC=CC=C5 |
| S21 | (2,5-diphenyl-2H-1,3,4-thiadiazol-3-yl)-phenylmethanone | C1=CC=C(C=C1)C2N(N=C(S2)C3=CC=CC=C3)C(=O)C4=CC=CC=C4 |
| S22 | 2-phenyl-1H-indole | C1=CC=C(C=C1)C2=CC3=CC=CC=C3N2 |
| S23 | 2-((phenylsulfonyl)methyl)-2,3,4,9-tetrahydro-1H-carbazole | S(=O)(=O)(C1(=CC=CC=C1))CC3(CC=2(NC=4(C=CC=CC=4(C=2CC3)))) |
| S24 | N-benzyl-N-(2,2-diphenylethyl)-3-phenoxypropan-1-amine | O(C1(=CC=CC=C1))CCCN(CC2(=CC=CC=C2))CC(C3(=CC=CC=C3))C4(=CC=CC=C4) |
| S25 | N-((1-phenylcyclohexyl)methyl)-1,2,3,4,4a,9,10,10a-octahydrophenanthrene-1-carboxamide | O=C(NCC2(C1(=CC=CC=C1))(CCCCC2))C5(C4(C(C3(=C(C=CC=C3)CC4))CCC5)) |
| S26 | N-phenylbenzenesulfonamide | C1=CC=C(C=C1)NS(=O)(=O)C2=CC=CC=C2 |
| S27 | N-(3-(benzo[d]isoxazol-6-yloxy)propyl)aniline | O1(N=CC3(=C1C=C(OCCCNC2(=CC=CC=C2))C=C3)) |
| S28 | 7-phenyl-3,4-dihydroquinolin-2(1H)-one | O=C3(NC1(=C(C=CC(=C1)C2(=CC=CC=C2))CC3)) |
| S29 | 4-(5,6,7,8-tetrahydronaphthalen-2-yl)-4,5,6,7-tetrahydrobenzofuran | O4(C3(=C(C(C2(=CC1(=C(CCCC1)C=C2)))CCC3)C=C4)) |
| S30 | N-phenylpyridin-2-amine | C1=CC=C(C=C1)NC2=CC=CC=N2 |
| S31 | 6H-indeno[1,2-c]isoquinoline-5,11-dione | C1=CC=C2C(=C1)C3=C(C4=CC=CC=C4C3=O)NC2=O |
| S32 | phenyl(5,6,7,8-tetrahydronaphthalen-2-yl)methanone | C1CCC2=C(C1)C=CC(=C2)C(=O)C3=CC=CC=C3 |
