# Supplementary material for: Finding the molecular scaffold of nuclear receptor inhibitors through high-throughput screening based on proteochemometric modelling
Source: J Cheminform. 2018 Apr 12;10:21. doi: 10.1186/s13321-018-0275-x (PMC5897275; doi:10.1186/s13321-018-0275-x)
Supplement: Supplementary file 8 — Additional file 8: Table S6. Data distribution of different NR targets. [file 13321_2018_275_MOESM8_ESM.docx]

Additional file 8: Table S6. Data distribution of different NR targets

| TargetID | Symbol | EC50 Number |
| --- | --- | --- |
| NR1C3 | PPAR_gamma | 2504 |
| NR1C1 | PPAR_alpha | 2275 |
| NR1C2 | PPAR_delta | 1120 |
| NR1H2 | LXR_beta | 382 |
| NR2B1 | RXR_alpha | 273 |
| NR1H3 | LXR_alpha | 205 |
| NR1H4 | FXR | 175 |
| NR2B3 | RXR_gamma | 156 |
| NR2B2 | RXR_beta | 142 |
| NR1D1 | Rev_erb_alpha | 23 |
| NR1I2 | PXR | 12 |
