# Supplementary material for: Finding the molecular scaffold of nuclear receptor inhibitors through high-throughput screening based on proteochemometric modelling
Source: J Cheminform. 2018 Apr 12;10:21. doi: 10.1186/s13321-018-0275-x (PMC5897275; doi:10.1186/s13321-018-0275-x)
Supplement: Supplementary file 9 — Additional file 9: Table S7. Information of crystal structure used for descriptor generation. [file 13321_2018_275_MOESM9_ESM.docx]

Additional file 9: Table S7. Information of crystal structure used for descriptor generation

| PDB ID | Symbol | Exp. Method | Resolution | Structure MW | Residue Count |
| --- | --- | --- | --- | --- | --- |
| 2Q3Y | NR0B2 | X-RAY DIFFRACTION | 2.4 | 30406.42 | 259 |
| 3UVV | NR1A1 | X-RAY DIFFRACTION | 2.95 | 58389.32 | 509 |
| 1DSZ | NR1B1 | X-RAY DIFFRACTION | 1.7 | 29654.19 | 201 |
| 3VI8 | NR1C1 | X-RAY DIFFRACTION | 1.75 | 31321.85 | 273 |
| 3TKM | NR1C2 | X-RAY DIFFRACTION | 1.95 | 31994.38 | 275 |
| 3U9Q | NR1C3 | X-RAY DIFFRACTION | 1.52 | 31877.6 | 278 |
| 1A6Y | NR1D1 | X-RAY DIFFRACTION | 2.3 | 34445.65 | 228 |
| 3CQV | NR1D2 | X-RAY DIFFRACTION | 1.9 | 23486.7 | 199 |
| 1N83 | NR1F1 | X-RAY DIFFRACTION | 1.63 | 31901.16 | 270 |
| 3L0L | NR1F3 | X-RAY DIFFRACTION | 1.74 | 61780.36 | 522 |
| 1UPV | NR1H2 | X-RAY DIFFRACTION | 2.1 | 29898.13 | 257 |
| 3IPQ | NR1H3 | X-RAY DIFFRACTION | 2 | 36351 | 308 |
| 1OSH | NR1H4 | X-RAY DIFFRACTION | 1.8 | 27583.96 | 232 |
| 1YNW | NR1I1 | X-RAY DIFFRACTION | 3 | 35710.27 | 245 |
| 3CTB | NR1I2 | X-RAY DIFFRACTION | 2 | 78368.8 | 688 |
| 3CBB | NR2A1 | X-RAY DIFFRACTION | 2 | 31260.2 | 198 |
| 2P1T | NR2B1 | X-RAY DIFFRACTION | 1.8 | 28816.56 | 253 |
| 1H9U | NR2B2 | X-RAY DIFFRACTION | 2.7 | 101130.9 | 896 |
| 2GL8 | NR2B3 | X-RAY DIFFRACTION | 2.4 | 107984.8 | 964 |
| 3P0U | NR2C2 | X-RAY DIFFRACTION | 3 | 56204.8 | 498 |
| 2EBL | NR2F1 | SOLUTION NMR | | 10031.11 | 89 |
| 3CJW | NR2F2 | X-RAY DIFFRACTION | 1.48 | 27311.7 | 244 |
| 3K6P | NR3B1 | X-RAY DIFFRACTION | 2 | 27586.98 | 248 |
| 1LO1 | NR3B2 | SOLUTION NMR | | 19223.21 | 124 |
| 2E2R | NR3B3 | X-RAY DIFFRACTION | 1.6 | 28115.87 | 244 |
| 2Z4J | NR3C4 | X-RAY DIFFRACTION | 2.6 | 30364.02 | 258 |
| 3V3E | NR4A1 | X-RAY DIFFRACTION | 2.06 | 57767.28 | 514 |
| 1OVL | NR4A2 | X-RAY DIFFRACTION | 2.2 | 186167.3 | 1626 |
| 1ZDT | NR5A1 | X-RAY DIFFRACTION | 2.1 | 59604.09 | 506 |
| 3PLZ | NR5A2 | X-RAY DIFFRACTION | 1.75 | 64028.82 | 542 |
