# Supplementary material for: Finding the molecular scaffold of nuclear receptor inhibitors through high-throughput screening based on proteochemometric modelling
Source: J Cheminform. 2018 Apr 12;10:21. doi: 10.1186/s13321-018-0275-x (PMC5897275; doi:10.1186/s13321-018-0275-x)
Supplement: Supplementary file 10 — Additional file 10: Table S8-1. Sequence similarity descriptors based on 30 NR proteins (T1). Table S8-2. Structure similarity descriptors based on 30 NR proteins (T2). [file 13321_2018_275_MOESM10_ESM.docx]

Additional file 10: Table S8-1. Sequence similarity descriptors based on 30 NR proteins (T1).

| T1 | NR0B2 | NR1A1 | NR1B1 | NR1C1 | NR1C2 | NR1C3 | NR1D1 | NR1D2 | NR1F1 | NR1F3 |
| --- | --- | --- | --- | --- | --- | --- | --- | --- | --- | --- |
| NR1C1 | 2.56 | 34.8 | 4.76 | 100 | 85.71 | 79.49 | 4.4 | 35.9 | 39.19 | 36.26 |
| NR1C2 | 2.55 | 37.82 | 4.73 | 85.09 | 100 | 79.27 | 4.36 | 38.91 | 47.27 | 39.64 |
| NR1C3 | 2.6 | 34.94 | 4.09 | 80.67 | 81.04 | 100 | 4.83 | 38.29 | 42.01 | 42.38 |
| NR1D1 | 6.38 | 11.7 | 65.96 | 15.96 | 12.77 | 13.83 | 100 | 7.45 | 14.89 | 12.77 |
| NR1H2 | 1.56 | 46.69 | 4.67 | 39.69 | 36.19 | 41.63 | 3.89 | 40.08 | 50.97 | 42.02 |
| NR1H3 | 2.12 | 49.47 | 3.53 | 43.82 | 38.16 | 40.64 | 2.83 | 43.11 | 51.24 | 47 |
| NR1H4 | 2.59 | 50.86 | 4.74 | 38.36 | 38.79 | 40.95 | 1.72 | 48.71 | 51.72 | 40.95 |
| NR1I2 | 1.45 | 34.01 | 3.2 | 25.58 | 29.65 | 30.81 | 1.45 | 30.81 | 36.92 | 31.69 |
| NR2B1 | 2.08 | 38.33 | 1.67 | 40.83 | 40 | 37.92 | 2.08 | 35.42 | 32.92 | 33.33 |
| NR2B2 | 2.23 | 38.84 | 1.79 | 40.63 | 41.52 | 39.29 | 2.68 | 38.84 | 35.27 | 32.59 |
| NR2B3 | 2.07 | 47.3 | 2.49 | 45.23 | 36.93 | 37.34 | 2.07 | 35.27 | 35.68 | 31.54 |
| T1 | **NR1H2** | **NR1H3** | **NR1H4** | **NR1I1** | **NR1I2** | **NR2A1** | **NR2B1** | **NR2B2** | **NR2B3** | **NR2C2** |
| NR1C1 | 37 | 46.15 | 32.6 | 2.2 | 32.6 | 5.49 | 35.9 | 33.33 | 39.93 | 32.6 |
| NR1C2 | 33.82 | 38.91 | 32.73 | 4 | 37.09 | 1.82 | 34.55 | 33.45 | 32.36 | 31.27 |
| NR1C3 | 40.15 | 42.75 | 35.32 | 5.58 | 39.03 | 3.35 | 33.83 | 32.71 | 33.46 | 31.23 |
| NR1D1 | 10.64 | 8.51 | 4.26 | 57.45 | 26.6 | 55.32 | 5.32 | 6.38 | 5.32 | 26.6 |
| NR1H2 | 100 | 83.66 | 43.97 | 2.72 | 56.03 | 6.23 | 46.69 | 40.47 | 41.63 | 31.52 |
| NR1H3 | 75.97 | 100 | 40.64 | 5.3 | 54.77 | 2.83 | 31.1 | 27.92 | 28.27 | 28.62 |
| NR1H4 | 48.71 | 49.14 | 100 | 6.9 | 47.84 | 2.59 | 38.36 | 38.36 | 44.4 | 34.05 |
| NR1I2 | 41.57 | 45.06 | 31.98 | 5.52 | 100 | 2.91 | 25.87 | 22.97 | 27.03 | 19.48 |
| NR2B1 | 50.42 | 36.67 | 37.08 | 7.5 | 36.25 | 3.33 | 100 | 87.08 | 94.17 | 49.17 |
| NR2B2 | 46.43 | 35.27 | 39.73 | 8.04 | 35.27 | 4.02 | 93.3 | 100 | 90.63 | 45.54 |
| NR2B3 | 44.4 | 33.2 | 43.15 | 7.47 | 39 | 3.73 | 93.78 | 84.23 | 100 | 43.57 |
| T1 | **NR2F1** | **NR2F2** | **NR3B1** | **NR3B2** | **NR3B3** | **NR3C4** | **NR4A1** | **NR4A2** | **NR5A1** | **NR5A2** |
| NR1C1 | 7.69 | 36.26 | 31.5 | 2.56 | 30.4 | 13.55 | 35.9 | 30.04 | 25.64 | 37.73 |
| NR1C2 | 2.18 | 33.45 | 36.73 | 7.64 | 30.18 | 32.36 | 39.64 | 36.36 | 26.18 | 30.18 |
| NR1C3 | 2.23 | 33.46 | 33.09 | 2.97 | 31.97 | 33.09 | 40.89 | 37.17 | 38.66 | 38.66 |
| NR1D1 | 58.51 | 35.11 | 10.64 | 61.7 | 5.32 | 11.7 | 8.51 | 7.45 | 8.51 | 23.4 |
| NR1H2 | 2.33 | 38.52 | 38.91 | 2.72 | 40.86 | 36.96 | 45.14 | 43.58 | 38.52 | 42.8 |
| NR1H3 | 2.83 | 39.22 | 35.34 | 3.18 | 41.34 | 32.51 | 44.88 | 44.52 | 35.34 | 41.7 |
| NR1H4 | 3.02 | 37.5 | 31.47 | 4.31 | 43.1 | 43.1 | 40.09 | 35.34 | 40.52 | 44.83 |
| NR1I2 | 2.62 | 31.4 | 22.67 | 3.2 | 28.49 | 26.45 | 29.65 | 34.59 | 26.45 | 35.76 |
| NR2B1 | 3.33 | 55.83 | 52.08 | 2.92 | 53.33 | 42.08 | 38.33 | 38.33 | 54.58 | 56.67 |
| NR2B2 | 4.91 | 56.25 | 52.68 | 3.13 | 52.68 | 43.75 | 37.05 | 37.05 | 47.77 | 52.23 |
| NR2B3 | 2.9 | 59.75 | 49.38 | 4.15 | 51.45 | 42.32 | 38.59 | 37.76 | 45.64 | 53.94 |

Supplementary Table 8-2. Structure similarity descriptors based on 30 NR proteins (T2).

| T2 | NR0B2 | NR1A1 | NR1B1 | NR1C1 | NR1C2 | NR1C3 | NR1D1 | NR1D2 | NR1F1 | NR1F3 |
| --- | --- | --- | --- | --- | --- | --- | --- | --- | --- | --- |
| NR1C1 | 20 | 38.13 | 14.12 | 100 | 83.02 | 77.27 | 13.16 | 50.77 | 40.93 | 37.31 |
| NR1C2 | 0 | 38.58 | 12.94 | 83.02 | 100 | 80.68 | 13.51 | 51.56 | 41.04 | 38.24 |
| NR1C3 | 20 | 41.25 | 16.9 | 77.27 | 80.68 | 100 | 15.79 | 51.78 | 44.79 | 41.15 |
| NR1D1 | 10 | 17.11 | 78.08 | 13.16 | 13.51 | 15.79 | 100 | 18.84 | 16.92 | 10.48 |
| NR1H2 | 10 | 47.72 | 16.9 | 39.85 | 37.31 | 40.7 | 18.67 | 53.57 | 44.94 | 40.49 |
| NR1H3 | 20 | 50.21 | 16.9 | 40.08 | 37.22 | 40.86 | 18.92 | 57.37 | 45.34 | 43.27 |
| NR1H4 | 40 | 40.57 | 4.92 | 36.33 | 32.83 | 38.28 | 22.03 | 52.51 | 44.13 | 36.59 |
| NR1I2 | 20 | 36.43 | 18.18 | 36.82 | 34.88 | 37.5 | 18.92 | 40.95 | 37.14 | 35 |
| NR2B1 | 0 | 40.85 | 22.22 | 32.68 | 35.36 | 37.11 | 25.35 | 44.67 | 35.27 | 33.06 |
| NR2B2 | 30 | 39.55 | 23.61 | 32.24 | 31.6 | 36.36 | 25.35 | 43.15 | 35.4 | 33.19 |
| NR2B3 | 30 | 38.46 | 23.94 | 31.84 | 34.17 | 33.06 | 12.05 | 48.28 | 36.16 | 33.48 |
| T2 | **NR1H2** | **NR1H3** | **NR1H4** | **NR1I1** | **NR1I2** | **NR2A1** | **NR2B1** | **NR2B2** | **NR2B3** | **NR2C2** |
| NR1C1 | 39.85 | 40.08 | 36.33 | 17.65 | 36.82 | 16.9 | 32.68 | 32.24 | 31.84 | 27.52 |
| NR1C2 | 37.31 | 37.22 | 32.83 | 14.78 | 34.88 | 15.38 | 35.36 | 31.6 | 34.17 | 26.74 |
| NR1C3 | 40.7 | 40.86 | 38.28 | 20.43 | 37.5 | 18.31 | 37.11 | 36.36 | 33.06 | 27.63 |
| NR1D1 | 18.67 | 18.92 | 22.03 | 68.06 | 18.92 | 67.57 | 25.35 | 25.35 | 12.05 | 21.05 |
| NR1H2 | 100 | 83.91 | 50.64 | 18.18 | 39.29 | 14.29 | 44.05 | 43.19 | 42.25 | 34.65 |
| NR1H3 | 83.91 | 100 | 53.81 | 10.53 | 39.43 | 14.08 | 40.64 | 41.87 | 39.32 | 33.03 |
| NR1H4 | 50.64 | 53.81 | 100 | 10.75 | 39.27 | 0 | 39.53 | 39.5 | 37.31 | 33.64 |
| NR1I2 | 39.29 | 39.43 | 39.27 | 10.26 | 100 | 14.29 | 32.73 | 32.95 | 31.52 | 25.85 |
| NR2B1 | 44.05 | 40.64 | 39.53 | 6 | 32.73 | 22.22 | 100 | 95.92 | 92.93 | 37.67 |
| NR2B2 | 43.19 | 41.87 | 39.5 | 11.29 | 32.95 | 13.7 | 95.92 | 100 | 89.29 | 43 |
| NR2B3 | 42.25 | 39.32 | 37.31 | 19.77 | 31.52 | 22.54 | 92.93 | 89.29 | 100 | 39.71 |
| T2 | **NR2F1** | **NR2F2** | **NR3B1** | **NR3B2** | **NR3B3** | **NR3C4** | **NR4A1** | **NR4A2** | **NR5A1** | **NR5A2** |
| NR1C1 | 16.67 | 28.4 | 28.24 | 15 | 33.85 | 26.32 | 36.43 | 34.11 | 31.56 | 31.8 |
| NR1C2 | 19.77 | 28.46 | 28.85 | 15 | 34.6 | 26.12 | 34.98 | 32.7 | 30.11 | 31.2 |
| NR1C3 | 18.6 | 29.96 | 33.73 | 17.5 | 36.82 | 30.8 | 40.39 | 37.25 | 31.42 | 33.98 |
| NR1D1 | 71.62 | 21.05 | 16 | 58.82 | 8.24 | 8.2 | 0 | 11.27 | 19.23 | 16.42 |
| NR1H2 | 16 | 37.55 | 42.11 | 14.86 | 45.34 | 38.59 | 42.19 | 42.8 | 35.1 | 37.6 |
| NR1H3 | 18.92 | 37.22 | 42.08 | 15.07 | 46.46 | 38.36 | 45.96 | 47.14 | 38.36 | 38.96 |
| NR1H4 | 13.56 | 34.74 | 37.73 | 9.52 | 38.96 | 33.77 | 38.24 | 39.57 | 32.63 | 35.17 |
| NR1I2 | 13.92 | 27.43 | 26.8 | 22.67 | 28.57 | 28.47 | 33.7 | 33.09 | 29.23 | 29.93 |
| NR2B1 | 16.25 | 48.34 | 45.29 | 12.73 | 52.68 | 38.79 | 38.72 | 41.23 | 50 | 51.97 |
| NR2B2 | 17.44 | 52.04 | 49.26 | 18.75 | 50.95 | 40.18 | 38.46 | 40.37 | 48.15 | 50.46 |
| NR2B3 | 9.2 | 51.02 | 44.5 | 18.99 | 49.29 | 38.81 | 39.37 | 40.74 | 47.69 | 49.09 |
