# Supplementary material for: Finding the molecular scaffold of nuclear receptor inhibitors through high-throughput screening based on proteochemometric modelling
Source: J Cheminform. 2018 Apr 12;10:21. doi: 10.1186/s13321-018-0275-x (PMC5897275; doi:10.1186/s13321-018-0275-x)
Supplement: Supplementary file 11 — Additional file 11: Table S9-1. Sequence similarity descriptors based on 5 NR proteins (T3). Table S9-2. Structure similarity descriptors based on 5 NR proteins (T4). [file 13321_2018_275_MOESM11_ESM.docx]

Additional file 11: Table S9-1. Sequence similarity descriptors based on 5 NR proteins (T3).

| T3 | | 3VI8 | 3TKM | 3U9Q | 1UPV | 2P1T |
| --- | --- | --- | --- | --- | --- | --- |
|  |  | NR1C1 | NR1C2 | NR1C3 | NR1H2 | NR2B1 |
| 3VI8 | NR1C1 | 100 | 85.71 | 79.49 | 37 | 35.9 |
| 3TKM | NR1C2 | 85.09 | 100 | 79.27 | 33.82 | 34.55 |
| 3U9Q | NR1C3 | 80.67 | 81.04 | 100 | 40.15 | 33.83 |
| 1A6Y | NR1D1 | 15.96 | 12.77 | 13.83 | 10.64 | 5.32 |
| 1UPV | NR1H2 | 39.69 | 36.19 | 41.63 | 100 | 46.69 |
| 3IPQ | NR1H3 | 43.82 | 38.16 | 40.64 | 75.97 | 31.1 |
| 1OSH | NR1H4 | 38.36 | 38.79 | 40.95 | 48.71 | 38.36 |
| 3CTB | NR1I2 | 25.58 | 29.65 | 30.81 | 41.57 | 25.87 |
| 2P1T | NR2B1 | 40.83 | 40 | 37.92 | 50.42 | 100 |
| 1H9U | NR2B2 | 40.63 | 41.52 | 39.29 | 46.43 | 93.3 |
| 2GL8 | NR2B3 | 45.23 | 36.93 | 37.34 | 44.4 | 93.78 |

Supplementary Table 9-2. Structure similarity descriptors based on 5 NR proteins (T4).

| T4 | | 3VI8 | 3TKM | 3U9Q | 1UPV | 2P1T |
| --- | --- | --- | --- | --- | --- | --- |
|  |  | NR1C1 | NR1C2 | NR1C3 | NR1H2 | NR2B1 |
| 3VI8 | NR1C1 | 100 | 83.02 | 77.27 | 39.85 | 32.68 |
| 3TKM | NR1C2 | 83.02 | 100 | 80.68 | 37.31 | 35.36 |
| 3U9Q | NR1C3 | 77.27 | 80.68 | 100 | 40.7 | 37.11 |
| 1A6Y | NR1D1 | 13.16 | 13.51 | 15.79 | 18.67 | 25.35 |
| 1UPV | NR1H2 | 39.85 | 37.31 | 40.7 | 100 | 44.05 |
| 3IPQ | NR1H3 | 40.08 | 37.22 | 40.86 | 83.91 | 40.64 |
| 1OSH | NR1H4 | 36.33 | 32.83 | 38.28 | 50.64 | 39.53 |
| 3CTB | NR1I2 | 36.82 | 34.88 | 37.5 | 39.29 | 32.73 |
| 2P1T | NR2B1 | 32.68 | 35.36 | 37.11 | 44.05 | 100 |
| 1H9U | NR2B2 | 32.24 | 31.6 | 36.36 | 43.19 | 95.92 |
| 2GL8 | NR2B3 | 31.84 | 34.17 | 33.06 | 42.25 | 92.93 |
