# Supplementary material for: Finding the molecular scaffold of nuclear receptor inhibitors through high-throughput screening based on proteochemometric modelling
Source: J Cheminform. 2018 Apr 12;10:21. doi: 10.1186/s13321-018-0275-x (PMC5897275; doi:10.1186/s13321-018-0275-x)
Supplement: Supplementary file 12 — Additional file 12: Table S10. Information of inhibitor descriptors. [file 13321_2018_275_MOESM12_ESM.docx]

Additional file 12: Table S10. Information of inhibitor descriptors.

| NO. | Name of descriptor | Category of descriptor | Reference |
| --- | --- | --- | --- |
| 1 | MaxEStateIndex | Basic EState definitions | Hall, Mohney and Kier. JCICS _31_ 76-81 (1991) |
| 2 | MinEStateIndex |  |  |
| 3 | MaxAbsEStateIndex |  |  |
| 4 | MinAbsEStateIndex |  |  |
| 5 | BalabanJ | Calculation of topological/topochemical descriptors. | Chem. Phys. Lett. vol 89, 399-404, (1982) |
| 6 | BertzCT | Calculation of topological/topochemical descriptors. | S. H. Bertz, J. Am. Chem. Soc., vol 103, 3599-3601 (1981) |
| 7 | Chi0 | Calculation of topological/topochemical descriptors. | Rev. Comput. Chem. 2:367-422 (1991) |
| 8 | Chi0n |  |  |
| 9 | Chi0v |  |  |
| 10 | Chi1 |  |  |
| 11 | Chi1n |  |  |
| 12 | Chi1v |  |  |
| 13 | Chi2n |  |  |
| 14 | Chi2v |  |  |
| 15 | Chi3n |  |  |
| 16 | Chi3v |  |  |
| 17 | Chi4n |  |  |
| 18 | Chi4v |  |  |
| 19 | EState_VSA1 | Hybrid EState-VSA descriptors (Estate VSA Descriptor 1-11) | http://www.rdkit.org/docs/api/rdkit.Chem.EState.EState_VSA-module.html |
| 20 | EState_VSA2 |  |  |
| 21 | EState_VSA3 |  |  |
| 22 | EState_VSA4 |  |  |
| 23 | EState_VSA5 |  |  |
| 24 | EState_VSA6 |  |  |
| 25 | EState_VSA7 |  |  |
| 26 | EState_VSA8 |  |  |
| 27 | EState_VSA9 |  |  |
| 28 | EState_VSA10 |  |  |
| 29 | EState_VSA11 |  |  |
| 30 | FractionCSP3 | Calculation of Lipinski parameters for molecules | http://www.rdkit.org/docs/api/rdkit.Chem.Lipinski-module.html#FractionCSP3 |
| 31 | HallKierAlpha | Calculation of topological/topochemical descriptors. | http://www.rdkit.org/docs/api/rdkit.Chem.GraphDescriptors-module.html#HallKierAlpha |
| 32 | HeavyAtomCount | Calculation of Lipinski parameters for molecules | http://www.rdkit.org/docs/api/rdkit.Chem.Lipinski-module.html#HeavyAtomCount |
| 33 | Ipc | Calculation of topological/topochemical descriptors. | D. Bonchev & N. Trinajstic, J. Chem. Phys. vol 67, 4517-4533 (1977 |
| 34 | Kappa1 | Calculation of topological/topochemical descriptors. | http://www.rdkit.org/docs/api/rdkit.Chem.GraphDescriptors-module.html |
| 35 | Kappa2 |  |  |
| 36 | Kappa3 |  |  |
| 37 | LabuteASA | Exposes functionality for MOE-like approximate molecular surface area descriptors | http://www.rdkit.org/docs/api/rdkit.Chem.MolSurf-module.html#LabuteASA |
| 38 | MolLogP | Atom-based calculation of LogP and MR using Crippen's approach | S. A. Wildman and G. M. Crippen JCICS 39 868-873 (1999) |
| 39 | MolMR |  |  |
| 40 | NHOHCount | Calculation of Lipinski parameters for molecules | http://www.rdkit.org/docs/api/rdkit.Chem.Lipinski-module.html |
| 41 | NOCount |  |  |
| 42 | NumAliphaticCarbocycles |  |  |
| 43 | NumAliphaticHeterocycles |  |  |
| 44 | NumAliphaticRings |  |  |
| 45 | NumAromaticCarbocycles |  |  |
| 46 | NumAromaticHeterocycles |  |  |
| 47 | NumAromaticRings |  |  |
| 48 | NumHAcceptors |  |  |
| 49 | NumHDonors |  |  |
| 50 | NumHeteroatoms |  |  |
| 51 | NumRotatableBonds |  |  |
| 52 | NumSaturatedCarbocycles |  |  |
| 53 | NumSaturatedHeterocycles |  |  |
| 54 | NumSaturatedRings |  |  |
| 55 | PEOE_VSA1 | Exposes functionality for MOE-like approximate molecular surface area descriptors (MOE Charge VSA Descriptor 1-14). | http://www.rdkit.org/docs/api/rdkit.Chem.MolSurf-module.html |
| 56 | PEOE_VSA2 |  |  |
| 57 | PEOE_VSA3 |  |  |
| 58 | PEOE_VSA4 |  |  |
| 59 | PEOE_VSA5 |  |  |
| 60 | PEOE_VSA6 |  |  |
| 61 | PEOE_VSA7 |  |  |
| 62 | PEOE_VSA8 |  |  |
| 63 | PEOE_VSA9 |  |  |
| 64 | PEOE_VSA10 |  |  |
| 65 | PEOE_VSA11 |  |  |
| 66 | PEOE_VSA12 |  |  |
| 67 | PEOE_VSA13 |  |  |
| 68 | PEOE_VSA14 |  |  |
| 69 | RingCount | Calculation of Lipinski parameters for molecules | http://www.rdkit.org/docs/api/rdkit.Chem.Lipinski-module.html#RingCount |
| 70 | SMR_VSA1 | Exposes functionality for MOE-like approximate molecular surface area descriptors (MOE MR VSA Descriptor 1-10). | http://www.rdkit.org/docs/api/rdkit.Chem.MolSurf-module.html |
| 71 | SMR_VSA2 |  |  |
| 72 | SMR_VSA3 |  |  |
| 73 | SMR_VSA4 |  |  |
| 74 | SMR_VSA5 |  |  |
| 75 | SMR_VSA6 |  |  |
| 76 | SMR_VSA7 |  |  |
| 77 | SMR_VSA8 |  |  |
| 78 | SMR_VSA9 |  |  |
| 79 | SMR_VSA10 |  |  |
| 80 | SlogP_VSA1 | Exposes functionality for MOE-like approximate molecular surface area descriptors (MOE logP VSA descriptor 1-12). | http://www.rdkit.org/docs/api/rdkit.Chem.MolSurf-module.html |
| 81 | SlogP_VSA2 |  |  |
| 82 | SlogP_VSA3 |  |  |
| 83 | SlogP_VSA4 |  |  |
| 84 | SlogP_VSA5 |  |  |
| 85 | SlogP_VSA6 |  |  |
| 86 | SlogP_VSA7 |  |  |
| 87 | SlogP_VSA8 |  |  |
| 88 | SlogP_VSA9 |  |  |
| 89 | SlogP_VSA10 |  |  |
| 90 | SlogP_VSA11 |  |  |
| 91 | SlogP_VSA12 |  |  |
| 92 | TPSA | Exposes functionality for MOE-like approximate molecular surface area descriptors. | http://www.rdkit.org/docs/api/rdkit.Chem.MolSurf-module.html#TPSA |
| 93 | VSA_EState1 | Hybrid EState-VSA descriptors (VSA Estate Descriptor 1-10) | http://www.rdkit.org/docs/api/rdkit.Chem.EState.EState_VSA-module.html |
| 94 | VSA_EState2 |  |  |
| 95 | VSA_EState3 |  |  |
| 96 | VSA_EState4 |  |  |
| 97 | VSA_EState5 |  |  |
| 98 | VSA_EState6 |  |  |
| 99 | VSA_EState7 |  |  |
| 100 | VSA_EState8 |  |  |
| 101 | VSA_EState9 |  |  |
| 102 | VSA_EState10 |  |  |
| 103 | fr_Al_COO | functions to match a bunch of fragment descriptors from a file | http://www.rdkit.org/docs/api/rdkit.Chem.Fragments-module.html#fr_Al_COO |
| 104 | fr_Al_OH | functions to match a bunch of fragment descriptors from a file | http://www.rdkit.org/docs/api/rdkit.Chem.Fragments-module.html#fr_Al_OH |
| 105 | fr_Al_OH_noTert | functions to match a bunch of fragment descriptors from a file | http://www.rdkit.org/docs/api/rdkit.Chem.Fragments-module.html#fr_Al_OH_noTert |
| 106 | fr_ArN | functions to match a bunch of fragment descriptors from a file | http://www.rdkit.org/docs/api/rdkit.Chem.Fragments-module.html#fr_ArN |
| 107 | fr_Ar_COO | functions to match a bunch of fragment descriptors from a file | http://www.rdkit.org/docs/api/rdkit.Chem.Fragments-module.html#fr_Ar_COO |
| 108 | fr_Ar_N | functions to match a bunch of fragment descriptors from a file | http://www.rdkit.org/docs/api/rdkit.Chem.Fragments-module.html#fr_Ar_N |
| 109 | fr_Ar_NH | functions to match a bunch of fragment descriptors from a file | http://www.rdkit.org/docs/api/rdkit.Chem.Fragments-module.html#fr_Ar_NH |
| 110 | fr_Ar_OH | functions to match a bunch of fragment descriptors from a file | http://www.rdkit.org/docs/api/rdkit.Chem.Fragments-module.html#fr_Ar_OH |
| 111 | fr_COO | functions to match a bunch of fragment descriptors from a file | http://www.rdkit.org/docs/api/rdkit.Chem.Fragments-module.html#fr_COO |
| 112 | fr_COO2 | functions to match a bunch of fragment descriptors from a file | http://www.rdkit.org/docs/api/rdkit.Chem.Fragments-module.html#fr_COO2 |
| 113 | fr_C_O | functions to match a bunch of fragment descriptors from a file | http://www.rdkit.org/docs/api/rdkit.Chem.Fragments-module.html#fr_C_O |
| 114 | fr_C_O_noCOO | functions to match a bunch of fragment descriptors from a file | http://www.rdkit.org/docs/api/rdkit.Chem.Fragments-module.html#fr_C_O_noCOO |
| 115 | fr_C_S | functions to match a bunch of fragment descriptors from a file | http://www.rdkit.org/docs/api/rdkit.Chem.Fragments-module.html#fr_C_S |
| 116 | fr_HOCCN | functions to match a bunch of fragment descriptors from a file | http://www.rdkit.org/docs/api/rdkit.Chem.Fragments-module.html#fr_HOCCN |
| 117 | fr_Imine | functions to match a bunch of fragment descriptors from a file | http://www.rdkit.org/docs/api/rdkit.Chem.Fragments-module.html#fr_Imine |
| 118 | fr_NH0 | functions to match a bunch of fragment descriptors from a file | http://www.rdkit.org/docs/api/rdkit.Chem.Fragments-module.html#fr_NH0 |
| 119 | fr_NH1 | functions to match a bunch of fragment descriptors from a file | http://www.rdkit.org/docs/api/rdkit.Chem.Fragments-module.html#fr_NH1 |
| 120 | fr_NH2 | functions to match a bunch of fragment descriptors from a file | http://www.rdkit.org/docs/api/rdkit.Chem.Fragments-module.html#fr_NH2 |
| 121 | fr_N_O | functions to match a bunch of fragment descriptors from a file | http://www.rdkit.org/docs/api/rdkit.Chem.Fragments-module.html#fr_N_O |
| 122 | fr_Ndealkylation1 | functions to match a bunch of fragment descriptors from a file | http://www.rdkit.org/docs/api/rdkit.Chem.Fragments-module.html#fr_Ndealkylation1 |
| 123 | fr_Ndealkylation2 | functions to match a bunch of fragment descriptors from a file | http://www.rdkit.org/docs/api/rdkit.Chem.Fragments-module.html#fr_Ndealkylation2 |
| 124 | fr_Nhpyrrole | functions to match a bunch of fragment descriptors from a file | http://www.rdkit.org/docs/api/rdkit.Chem.Fragments-module.html#fr_Nhpyrrole |
| 125 | fr_SH | functions to match a bunch of fragment descriptors from a file | http://www.rdkit.org/docs/api/rdkit.Chem.Fragments-module.html#fr_SH |
| 126 | fr_aldehyde | functions to match a bunch of fragment descriptors from a file | http://www.rdkit.org/docs/api/rdkit.Chem.Fragments-module.html#fr_aldehyde |
| 127 | fr_alkyl_carbamate | functions to match a bunch of fragment descriptors from a file | http://www.rdkit.org/docs/api/rdkit.Chem.Fragments-module.html#fr_alkyl_carbamate |
| 128 | fr_alkyl_halide | functions to match a bunch of fragment descriptors from a file | http://www.rdkit.org/docs/api/rdkit.Chem.Fragments-module.html#fr_alkyl_halide |
| 129 | fr_allylic_oxid | functions to match a bunch of fragment descriptors from a file | http://www.rdkit.org/docs/api/rdkit.Chem.Fragments-module.html#fr_allylic_oxid |
| 130 | fr_amide | functions to match a bunch of fragment descriptors from a file | http://www.rdkit.org/docs/api/rdkit.Chem.Fragments-module.html#fr_amide |
| 131 | fr_amidine | functions to match a bunch of fragment descriptors from a file | http://www.rdkit.org/docs/api/rdkit.Chem.Fragments-module.html#fr_amidine |
| 132 | fr_aniline | functions to match a bunch of fragment descriptors from a file | http://www.rdkit.org/docs/api/rdkit.Chem.Fragments-module.html#fr_aniline |
| 133 | fr_aryl_methyl | functions to match a bunch of fragment descriptors from a file | http://www.rdkit.org/docs/api/rdkit.Chem.Fragments-module.html#fr_aryl_methyl |
| 134 | fr_azide | functions to match a bunch of fragment descriptors from a file | http://www.rdkit.org/docs/api/rdkit.Chem.Fragments-module.html#fr_azide |
| 135 | fr_azo | functions to match a bunch of fragment descriptors from a file | http://www.rdkit.org/docs/api/rdkit.Chem.Fragments-module.html#fr_azo |
| 136 | fr_barbitur | functions to match a bunch of fragment descriptors from a file | http://www.rdkit.org/docs/api/rdkit.Chem.Fragments-module.html#fr_barbitur |
| 137 | fr_benzene | functions to match a bunch of fragment descriptors from a file | http://www.rdkit.org/docs/api/rdkit.Chem.Fragments-module.html#fr_benzene |
| 138 | fr_benzodiazepine | functions to match a bunch of fragment descriptors from a file | http://www.rdkit.org/docs/api/rdkit.Chem.Fragments-module.html#fr_benzodiazepine |
| 139 | fr_bicyclic | functions to match a bunch of fragment descriptors from a file | http://www.rdkit.org/docs/api/rdkit.Chem.Fragments-module.html#fr_bicyclic |
| 140 | fr_diazo | functions to match a bunch of fragment descriptors from a file | http://www.rdkit.org/docs/api/rdkit.Chem.Fragments-module.html#fr_diazo |
| 141 | fr_dihydropyridine | functions to match a bunch of fragment descriptors from a file | http://www.rdkit.org/docs/api/rdkit.Chem.Fragments-module.html#fr_dihydropyridine |
| 142 | fr_epoxide | functions to match a bunch of fragment descriptors from a file | http://www.rdkit.org/docs/api/rdkit.Chem.Fragments-module.html#fr_epoxide |
| 143 | fr_ester | functions to match a bunch of fragment descriptors from a file | http://www.rdkit.org/docs/api/rdkit.Chem.Fragments-module.html#fr_ester |
| 144 | fr_ether | functions to match a bunch of fragment descriptors from a file | http://www.rdkit.org/docs/api/rdkit.Chem.Fragments-module.html#fr_ether |
| 145 | fr_furan | functions to match a bunch of fragment descriptors from a file | http://www.rdkit.org/docs/api/rdkit.Chem.Fragments-module.html#fr_furan |
| 146 | fr_guanido | functions to match a bunch of fragment descriptors from a file | http://www.rdkit.org/docs/api/rdkit.Chem.Fragments-module.html#fr_guanido |
| 147 | fr_halogen | functions to match a bunch of fragment descriptors from a file | http://www.rdkit.org/docs/api/rdkit.Chem.Fragments-module.html#fr_halogen |
| 148 | fr_hdrzine | functions to match a bunch of fragment descriptors from a file | http://www.rdkit.org/docs/api/rdkit.Chem.Fragments-module.html#fr_hdrzine |
| 149 | fr_hdrzone | functions to match a bunch of fragment descriptors from a file | http://www.rdkit.org/docs/api/rdkit.Chem.Fragments-module.html#fr_hdrzone |
| 150 | fr_imidazole | functions to match a bunch of fragment descriptors from a file | http://www.rdkit.org/docs/api/rdkit.Chem.Fragments-module.html#fr_imidazole |
| 151 | fr_imide | functions to match a bunch of fragment descriptors from a file | http://www.rdkit.org/docs/api/rdkit.Chem.Fragments-module.html#fr_imide |
| 152 | fr_isocyan | functions to match a bunch of fragment descriptors from a file | http://www.rdkit.org/docs/api/rdkit.Chem.Fragments-module.html#fr_isocyan |
| 153 | fr_isothiocyan | functions to match a bunch of fragment descriptors from a file | http://www.rdkit.org/docs/api/rdkit.Chem.Fragments-module.html#fr_isothiocyan |
| 154 | fr_ketone | functions to match a bunch of fragment descriptors from a file | http://www.rdkit.org/docs/api/rdkit.Chem.Fragments-module.html#fr_ketone |
| 155 | fr_ketone_Topliss | functions to match a bunch of fragment descriptors from a file | http://www.rdkit.org/docs/api/rdkit.Chem.Fragments-module.html#fr_ketone_Topliss |
| 156 | fr_lactam | functions to match a bunch of fragment descriptors from a file | http://www.rdkit.org/docs/api/rdkit.Chem.Fragments-module.html#fr_lactam |
| 157 | fr_lactone | functions to match a bunch of fragment descriptors from a file | http://www.rdkit.org/docs/api/rdkit.Chem.Fragments-module.html#fr_lactone |
| 158 | fr_methoxy | functions to match a bunch of fragment descriptors from a file | http://www.rdkit.org/docs/api/rdkit.Chem.Fragments-module.html#fr_methoxy |
| 159 | fr_morpholine | functions to match a bunch of fragment descriptors from a file | http://www.rdkit.org/docs/api/rdkit.Chem.Fragments-module.html#fr_morpholine |
| 160 | fr_nitrile | functions to match a bunch of fragment descriptors from a file | http://www.rdkit.org/docs/api/rdkit.Chem.Fragments-module.html#fr_nitrile |
| 161 | fr_nitro | functions to match a bunch of fragment descriptors from a file | http://www.rdkit.org/docs/api/rdkit.Chem.Fragments-module.html#fr_nitro |
| 162 | fr_nitro_arom | functions to match a bunch of fragment descriptors from a file | http://www.rdkit.org/docs/api/rdkit.Chem.Fragments-module.html#fr_nitro_arom |
| 163 | fr_nitro_arom_nonortho | functions to match a bunch of fragment descriptors from a file | http://www.rdkit.org/docs/api/rdkit.Chem.Fragments-module.html#fr_nitro_arom_nonortho |
| 164 | fr_nitroso | functions to match a bunch of fragment descriptors from a file | http://www.rdkit.org/docs/api/rdkit.Chem.Fragments-module.html#fr_nitroso |
| 165 | fr_oxazole | functions to match a bunch of fragment descriptors from a file | http://www.rdkit.org/docs/api/rdkit.Chem.Fragments-module.html#fr_oxazole |
| 166 | fr_oxime | functions to match a bunch of fragment descriptors from a file | http://www.rdkit.org/docs/api/rdkit.Chem.Fragments-module.html#fr_oxime |
| 167 | fr_para_hydroxylation | functions to match a bunch of fragment descriptors from a file | http://www.rdkit.org/docs/api/rdkit.Chem.Fragments-module.html#fr_para_hydroxylation |
| 168 | fr_phenol | functions to match a bunch of fragment descriptors from a file | http://www.rdkit.org/docs/api/rdkit.Chem.Fragments-module.html#fr_phenol |
| 169 | fr_phenol_noOrthoHbond | functions to match a bunch of fragment descriptors from a file | http://www.rdkit.org/docs/api/rdkit.Chem.Fragments-module.html#fr_phenol_noOrthoHbond |
| 170 | fr_phos_acid | functions to match a bunch of fragment descriptors from a file | http://www.rdkit.org/docs/api/rdkit.Chem.Fragments-module.html#fr_phos_acid |
| 171 | fr_phos_ester | functions to match a bunch of fragment descriptors from a file | http://www.rdkit.org/docs/api/rdkit.Chem.Fragments-module.html#fr_phos_ester |
| 172 | fr_piperdine | functions to match a bunch of fragment descriptors from a file | http://www.rdkit.org/docs/api/rdkit.Chem.Fragments-module.html#fr_piperdine |
| 173 | fr_piperzine | functions to match a bunch of fragment descriptors from a file | http://www.rdkit.org/docs/api/rdkit.Chem.Fragments-module.html#fr_piperzine |
| 174 | fr_priamide | functions to match a bunch of fragment descriptors from a file | http://www.rdkit.org/docs/api/rdkit.Chem.Fragments-module.html#fr_priamide |
| 175 | fr_prisulfonamd | functions to match a bunch of fragment descriptors from a file | http://www.rdkit.org/docs/api/rdkit.Chem.Fragments-module.html#fr_prisulfonamd |
| 176 | fr_pyridine | functions to match a bunch of fragment descriptors from a file | http://www.rdkit.org/docs/api/rdkit.Chem.Fragments-module.html#fr_pyridine |
| 177 | fr_quatN | functions to match a bunch of fragment descriptors from a file | http://www.rdkit.org/docs/api/rdkit.Chem.Fragments-module.html#fr_quatN |
| 178 | fr_sulfide | functions to match a bunch of fragment descriptors from a file | http://www.rdkit.org/docs/api/rdkit.Chem.Fragments-module.html#fr_sulfide |
| 179 | fr_sulfonamd | functions to match a bunch of fragment descriptors from a file | http://www.rdkit.org/docs/api/rdkit.Chem.Fragments-module.html#fr_sulfonamd |
| 180 | fr_sulfone | functions to match a bunch of fragment descriptors from a file | http://www.rdkit.org/docs/api/rdkit.Chem.Fragments-module.html#fr_sulfone |
| 181 | fr_term_acetylene | functions to match a bunch of fragment descriptors from a file | http://www.rdkit.org/docs/api/rdkit.Chem.Fragments-module.html#fr_term_acetylene |
| 182 | fr_tetrazole | functions to match a bunch of fragment descriptors from a file | http://www.rdkit.org/docs/api/rdkit.Chem.Fragments-module.html#fr_tetrazole |
| 183 | fr_thiazole | functions to match a bunch of fragment descriptors from a file | http://www.rdkit.org/docs/api/rdkit.Chem.Fragments-module.html#fr_thiazole |
| 184 | fr_thiocyan | functions to match a bunch of fragment descriptors from a file | http://www.rdkit.org/docs/api/rdkit.Chem.Fragments-module.html#fr_thiocyan |
| 185 | fr_thiophene | functions to match a bunch of fragment descriptors from a file | http://www.rdkit.org/docs/api/rdkit.Chem.Fragments-module.html#fr_thiophene |
| 186 | fr_unbrch_alkane | functions to match a bunch of fragment descriptors from a file | http://www.rdkit.org/docs/api/rdkit.Chem.Fragments-module.html#fr_unbrch_alkane |
| 187 | fr_urea | functions to match a bunch of fragment descriptors from a file | http://www.rdkit.org/docs/api/rdkit.Chem.Fragments-module.html#fr_urea |
